# Revisiting the Surface Brightness Profile of the Stellar Disk with the Statistical Mechanics of the Self-Gravitating System with the Central Body

**DOI:** 10.3390/e26040297

**Published:** 2024-03-28

**Authors:** Dong-Biao Kang

**Affiliations:** 1 School of Intelligent Manufacturing, Zhejiang Guangsha Vocational and Technological University of Construction, Jinhua 322100, China; dbkang@zjgsdx.edu.cn; 2 Institute of Theoretical Physics, Chinese Academy of Sciences, Beijing 100190, China

**Keywords:** stellar disk, black hole, surface brightness profile, self-gravitating system, statistical mechanics

## Abstract

We have explored the exponential surface brightness profile (SBP) of stellar disks, a topic extensively discussed by many authors yet seldom integrated with the study of correlations between black holes, bulges, and entire disks. Building upon our prior work in the statistical mechanics of disk-shaped systems and aligning with methodologies from other research, we analyze the influence of the central body. This analysis reveals analytical relationships among black holes, bulges, and the entire stellar disk. Additionally, we incorporate a specific angular momentum distribution (SAMD) that aligns more closely with observational data, showing that for the self-gravitating disk, with the same surface density, a reduction in its spin results in only a slight decrease in its radius, whereas with the same SAMD, an increment in its spin significantly limits its extent. A key feature of our model is its prediction that the surface density profile of an isolated disk will invariably exhibit downbending at a sufficient distance, a hypothesis that future observations can test. Our refined equations provide a notably improved fit for SBPs, particularly in the central regions of stellar disks. While our findings underscore the significance of statistical mechanics in comprehending spiral galaxy structures, they also highlight areas in our approach that warrant further discussion and exploration.

## 1. Introduction

Spiral galaxies exhibit a multitude of intriguing properties. For instance, the SBPs of their stellar disks often conform to an exponential law. Early research by [1,2,3] attributed this phenomenon to the conservation of angular momentum distribution. Subsequently, [4] highlighted the significance of stellar radial motions in influencing these characteristics. Further, [5,6] demonstrated that stellar scattering by massive clumps could give rise to exponential disks. Ref. [7] also underscored the role of magnetic stress as a viscosity source aiding star formation. Additionally, the central supermassive black hole in these galaxies shows a correlation with both the bulge and the entire stellar disk. This is exemplified by the $M_{•}-M_{b}$ relationship, as discussed in [8,9,10], and the $M_{•}-\sigma$ relationship proposed by [11], where $M_{•}$ and $M_{b}$ represent the masses of the black hole and bulge, respectively, and σ is the velocity dispersion of the stars in the bulge. Although many authors, such as [12,13,14,15], suggest that these correlations may stem from black hole accretion and feedback mechanisms, the underlying physical connections among the black hole, bulge, and disk are yet to be comprehensively understood.

Among these works, it is noteworthy that the statistical mechanics of self-gravitating systems may provide a unique perspective on the aforementioned issues. This approach was pioneered by [16], who examined the equilibrium state of collisionless self-gravitating systems using the maximum entropy principle under mass and energy constraints. Despite its potential, this method encounters several challenges, such as non-ergodicity, ensemble inequivalence, and non-extensive energy, as detailed in [17,18]. In the context of disk-shaped systems, [19] introduced a local velocity distribution using the maximum entropy approach. Additionally, [20] proposed steady-state distribution functions derived from minimal entropy gradients. Works by [21,22] demonstrate that exponential disks can be achieved by maximizing entropy subject to mass and angular momentum constraints. Additionally, based on the saddle point entropy concept introduced by [23], we have derived an equilibrium density profile of a spherical self-gravitating system with finite mass for the first time [24], and we believe it can also provide unique insights on the disk-shaped self-gravitating system [25]. However, these studies have not concurrently considered the exponential disk and the black hole.

Ref. [26] considers the maximized entropy with the central body for the spherical system. In this work, based on our previous work [25], we will study the effects of the central black hole on the stellar disk. This paper is structured as follows: Section 2 provides a review of our earlier work to establish the foundational concepts. In Section 3, we delve into the impact of the central black hole on the stellar disk, examining scenarios both with and without the inclusion of angular momentum. This analysis aims to elucidate the intricate relationships among the black hole, the bulge, and the disk of the galaxy. Finally, in the concluding section (Section 4), we juxtapose our characteristic findings with other studies, highlighting the unique aspects of our model. Furthermore, we discuss existing limitations and outline potential areas for future research to address these challenges.

## 2. Entropy Principle

Let us first review our previous work [25]. By virtue of the large radial-to-vertical scale ratio of the disk [27] and for the purpose of simplifying the issue, we calculate in two dimensions, which is also adopted by other authors [21]. This can also be consistent with the flat rotation curve because the circular velocity is

(1) $$v_{c}^{2}=R\frac{\partial \Phi }{\partial R}\propto R\frac{dlnR}{dR}=const,\mathrm{for}\;R\to \infty$$

where Φ is the gravitational potential of the disk. We assume the coarse-grained phase space distribution can be written as

(2) $$f\left(R,v\right)=A\left(R\right)e^{-\frac{v^{2}}{4\sigma {\left(R\right)}^{2}}}$$

where $\sigma^{2}$ is the half of velocity dispersion with the assumption of the isotropic velocity dispersion $\sigma_{t}^{2}=\sigma_{R}^{2}+\sigma_{\phi }^{2}=2\sigma^{2}$, *A* is a function of *R*, and (2) means that the local velocity distribution is Gaussian and fundamentally consistent with the work of [19,28]. We define ρ and $P=\rho \sigma^{2}$ to be the disk’s surface density and pressure, respectively, and they are related to A(R) by $\rho \left(R\right)=\int fd^{2}v$. Then, the Boltzmann–Gibbs entropy will be

(3) $$S=-\int f\ln fd^{2}xd^{2}v=\int dRR\rho \ln\left(\frac{P(R)}{\rho {\left(R\right)}^{2}}\right)$$

The Poisson equation is

(4) $$\frac{1}{R}\frac{\partial }{\partial R}R\frac{\partial \Phi }{\partial R}=2\pi G\rho \left(R\right).$$

The potential energy is [27]

(5) $$E=-\int dRR\rho \left(R\right)R\frac{\partial \Phi }{\partial R}.$$

To calculate the variation equation:

(6) $$\delta S_{t}=\delta S-\alpha \delta M-\beta \delta E=0,$$

we denote y(R)=R∂Φ(R)/∂R, then

(7) $$S=\int dRy^{\prime }\left(R\right)\ln\frac{P}{{\left(y^{\prime }\left(R\right)/R\right)}^{2}},$$

(8) $$M=y\left(\infty \right),\;E=-\frac{1}{2\pi G}\int dRy^{\prime }\left(R\right)y\left(R\right),$$

which are obtained by (4) and (5). Note that the constant *G* in this work is the gravitational constant in 2D gravity, which is different from the usual value in 3D Newtonian gravity. Here, we only constrained the potential energy because it is proportional to the total energy by the virial theorem. $S_{t}$ is the functional of *y* and $y^{\prime }$, and its variation can be calculated by (see [23])

$$\frac{\partial S_{t}}{\partial y}-{\left(\frac{\partial S_{t}}{\partial y^{\prime }}\right)}^{\prime }=0$$

where the apostrophe (′) means the derivative with *R*. Then, we have

(9) $$\rho \left(R\right)=\alpha P{\left(R\right)}^{1/2}$$

where α can be determined by the mass of the disk $\alpha =\alpha (M_{d})$. The dynamical equilibrium equation for the disk is [27]:

(10) $$\frac{\partial P}{\partial R}+\frac{\rho }{R}{\bar{v_{\phi }}}^{2}=-\rho \frac{\partial \Phi }{\partial R},$$

which has made the assumption of zero mixed moments. Note that the energy constraint β has disappeared, which is also faced by [21] and will be further discussed in the last section; the mass constraint is transformed into a boundary condition by virtue of (8).

## 3. Effect of the Central Body

**Figure 1:**
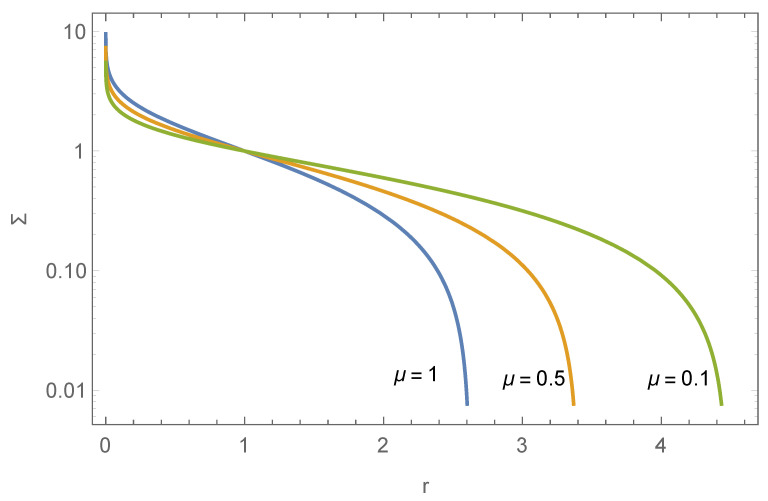
The influence of μ on the surface density profile of the stellar disk, as described by Equation (14). We choose Σ(1)=p(1)=f(1)=γ=1.

We first study the case without constraining the angular momentum. To examine the influence of a black hole, we adopt a methodology similar to [26]. This involves modifying the gravitational potential in our calculations to $\Phi_{t}=\Phi +\Phi_{•}$, where $\Phi_{•}=GM_{•}\ln R$ represents the gravitational potential generated by the central body with mass $M_{•}$. In the central region, where angular momentum may be disregarded, we assume $\bar{v_{\phi }}=0$. Additionally, we exclude material exchange between the central body and the disk. Through a repetition of the variation process described in the previous section, we derive the following set of equations:

(11) $$\begin{array}{c} \rho \left(R\right)=\alpha P{\left(R\right)}^{1/2},\\ F^{\prime }\left(R\right)+\frac{F(R)}{R}=2\pi G\rho \left(R\right),\\ P^{\prime }\left(R\right)=-\rho \left(R\right)\left(F\left(R\right)+\frac{GM_{•}}{R}\right), \end{array}$$

where F(R)=∂Φ/∂R and the last equation differs from those in the previous section. If the $M_{•}$-related term is negligible, ρ will satisfy:

(12) $$\rho^{\prime \prime }\left(R\right)+\frac{\rho^{\prime }\left(R\right)}{R}=-\pi G\alpha^{2}\rho \left(R\right),$$

and if α=0,

(13) $$\rho =\rho_{0}\ln\left(\frac{R_{d}}{R}\right),$$

where $R_{d}$ denotes the disk radius and $\rho_{0}$ the central density. If α≠0, ρ is a linear combination of the first kind of Bessel J function and the second kind of Bessel function. The numerical solution, as discussed in [25], shows that ρ(R) can still be well approximated by the logarithmic form (13). This logarithmic function, inverse to the exponential function, depicts a straight line over certain intervals in surface brightness profile plots, leading us to propose the log model for describing the downbending exponential profile. Conversely, if the $M_{•}$ related term predominates in the last equation of (11), particularly in the central region, ρ(R) will still be proportional to −lnR, implying a central surface density slope tending towards zero, unaffected by the black hole. To render each quantity dimensionless, we recast Equation (11) as follows:

(14) $$\begin{array}{c} \Sigma \left(r\right)=p{\left(r\right)}^{1/2},\\ \frac{f(r)}{r}+\frac{df(r)}{dr}=\nu \Sigma \left(r\right),\\ \frac{dp(r)}{dr}=-\Sigma \left(r\right)\left(f\left(r\right)+\frac{\mu }{r}\right), \end{array}$$

where $r=R/R_{*}$, $p=P/P_{*}$, $\Sigma =\rho /\rho_{*}$, $P_{*}=P\left(R_{*}\right)$, $\nu =2\pi G\frac{\rho_{*}^{2}R_{*}^{2}}{P_{*}}$, $\mu =\frac{GM_{•}\rho_{*}}{P_{*}}$, and f(r) is the dimensionless quantity corresponding to F(R). Here, $\rho \left(R_{*}\right)=\rho_{*}$, $P_{*}=P\left(R_{*}\right)$, and $R_{*}$ is a fixed scale. ν can be rescaled to 1, and μ is the only shape parameter. The boundary condition is set as Σ(1)=1, p(1)=1, and f(1) determined by the disk’s mass constraint $f\left(1\right)=f{\left(1\right)}_{M=M_{d}}$, and then Equation (14) can be solved numerically. The impact of the black hole is illustrated in Figure 1.

With the above method, few disks’ central SBPs can be fitted well. Many disks’ central SBPs are much steeper than the logarithmic profile. We hypothesize that this discrepancy may arise in many disks where $M_{•}$ is relatively larger, and $\Phi_{•}=GM_{•}\ln R$ may not adequately describe the effect of the black hole. To address this issue, we propose two solutions. In the first approach, we persist with 2D calculations and redefine $\Phi_{•}$ as $\Phi_{•}=-\frac{GM_{•}R_{h}}{R}$, introducing a constant scale $R_{h}$ to balance dimensions. Subsequently, we reevaluate Equation (6), leading to the following adjusted set of equations:

(15) $$\begin{array}{ccc} \frac{d\ln p}{dr}-\frac{2d\ln\Sigma }{dr} & = & \frac{b}{r^{2}},\\ \frac{f(r)}{r}+\frac{df(r)}{dr} & = & \Sigma (r),\\ \frac{dp(r)}{dr} & = & -\Sigma \left(r\right)\left(f\left(r\right)+\frac{\mu }{r^{2}}\right) \end{array}$$

Here, $b=\beta \frac{GM_{•}R_{h}}{R_{*}}$ and $\mu =\frac{GM_{•}R_{h}\rho_{*}}{R_{*}P_{*}}$, with other definitions consistent with those previously stated. Note that β is determined by the energy constraint. Analyzing Equation (15), we find that in the central region, if the black hole mass is large (but not excessively so), terms involving *b* and f(r) can be ignored, while the μ-related term remains significant. This leads to a solution for (15):

(16) $$\Sigma \left(r\right)=\frac{\mu }{2r},$$

indicating a cusp formation, which we speculate may collapse into a spherical bulge. The total mass of this bulge is given by:

(17) $$M_{b}=\rho_{*}R_{*}^{2}\times 2\pi \int_{0}^{\frac{R_{b}^{\prime }}{R_{*}}}\Sigma \left(r\right)rdr=\pi GM_{•}R_{h}R_{b}^{\prime }\frac{\rho_{*}^{2}}{P_{*}}.$$

The radius of the pre-bulge cusp region, $R_{b}^{\prime }$, differs from the bulge’s radius and is estimated by:

(18) $$\frac{GM_{•}R_{h}}{R_{b}^{2}}∼F\left(R_{b}^{\prime }\right)=\frac{GM_{b}}{R_{b}^{\prime }},$$

leading to:

(19) $$R_{b}^{\prime }∼\frac{M_{•}}{M_{b}}R_{h}.$$

Combining Equations (17) and (19), we obtain:

(20) $$R_{b}^{\prime }=\sqrt{\frac{P_{*}}{\pi G\rho_{*}^{2}}},$$

and

(21) $$\frac{M_{•}}{M_{b}}=\sqrt{\frac{P_{*}}{\pi GR_{h}^{2}\rho_{*}^{2}}}.$$

Equation (21) suggests a correlation among $M_{b}$, $M_{•}$, and the overall disk properties, warranting comparison with observational data. It is important to note that in this analysis, we do not consider the disk mass accreted by the black hole or the feedback mechanism. Moreover, $R_{h}$ is introduced solely for dimensional consistency in 2D calculations, aligning with the flat rotation curve hypothesis. Therefore, we propose $R_{h}$ to represent the scale of the disk’s host dark matter halo. If the right-hand side of Equation (21) remains relatively constant across different disks, it implies a fundamental constancy in the mass ratio between the black hole and the bulge, aligning with findings by [29].

**Figure 2:**
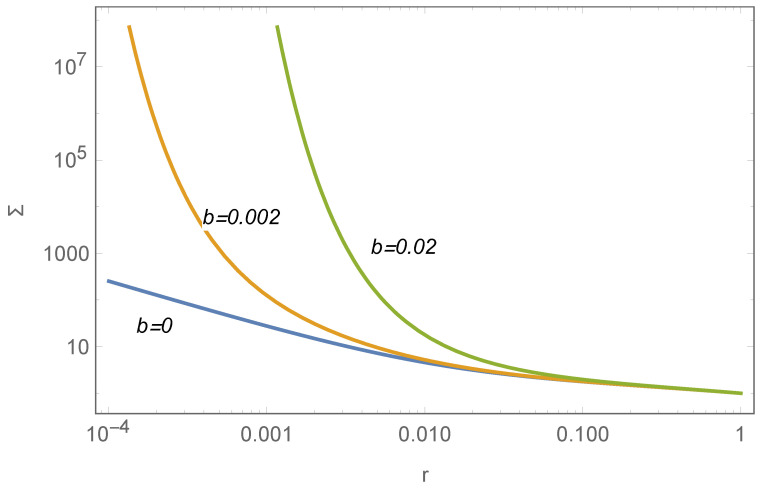
The influence of *b* on the surface density profile of the stellar disk, governed by Equation (15). Parameters set as Σ(1)=p(1)=f(1)=1 and μ=0.05.

**Figure 3:**
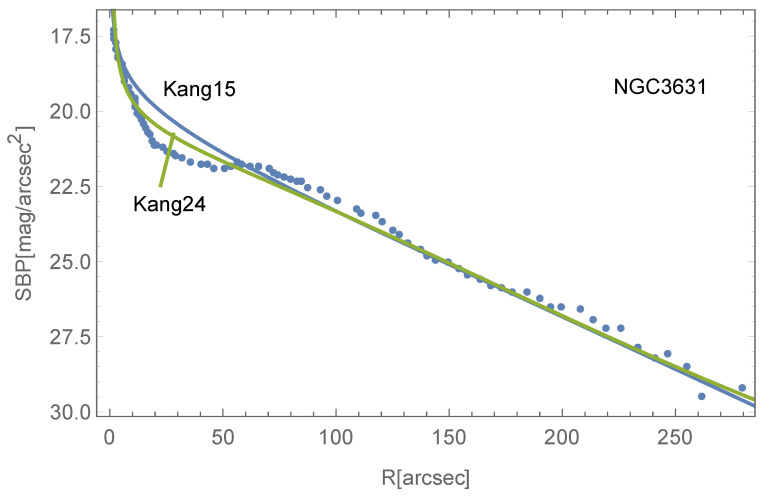
The SBP of NGC3631 (cited from [30]) fitted by equations in [25] (Kang15) and Equation (28) in this work (Kang24). The root mean squared error of Kang15 and Kang24 is 0.565 and 0.351, respectively.

It is plausible that the solution with b=0, under the following angular momentum constraint, aligns well with the SBPs of many stellar disks. However, there are exceptions characterized by a very steep central slope, necessitating the consideration of cases where b≠0. In such scenarios, $M_{•}$ is significantly larger, complicating the analytical solution of Equation (15). The numerical solution suggests a surface density profile in the central region described by:

(22) $$\Sigma \left(r\right)\propto \frac{1}{\sqrt{\mu b}}e^{\frac{b}{r}},$$

indicating a much steeper slope. Figure 2 illustrates its numerical solution, yet deriving an analytical relationship between $M_{b}$ and $M_{•}$ remains challenging. The influence of b≠0 appears predominantly in the central region of the disk, likely having minimal impact on $M_{b}$. Consequently, Equation (21) may still be applicable. An observational example with b≠0 is shown in Figure 3.

Another approach to address the steep central slope involves revisiting the variation method in 3D. Observations of these disks [30] often show pits between bulges and disks, hinting at a potential gap, possibly resulting from a cusp collapse forming the bulge. If the mass of the central body is substantial, this gap could be more pronounced, leading to a degree of isolation between the bulge and the disk. As an approximation, we can model the central body and the bulge as an isolated 3D system. Ref. [27], on page 336, demonstrates that an isothermal bulge can lead to a central body density profile $\propto 1/r^{3/2}$, steeper than $r^{-1}$. If we apply our method into the 3D spherical system, we can have:

(23) $$\begin{array}{ccc} \frac{d\ln p}{dr}-\frac{5d\ln\rho }{3dr} & = & \frac{b\mu }{r^{2}}+bf\left(r\right),\\ \frac{f(r)}{r}+\frac{2df(r)}{dr} & = & \rho (r),\\ \frac{dp(r)}{dr} & = & -\rho \left(r\right)\left(f\left(r\right)+\frac{\mu }{r^{2}}\right), \end{array}$$

where *r* denotes the 3D radius in this context. In the central region, we can approximate $\rho \left(r\right)\propto \exp\left(r_{0}/r\right)$ where $r_{0}\propto b\mu$. This cusp is consistent with both the initial approach and the findings of [26], even though it requires converting volume density to surface density. However, this model cannot describe the entire disk, and hence, our subsequent analyses will continue with the initial method.

However, the realistic stellar disk at equilibrium always has the finite angular momentum, and we need to study the case with this constraint. In the work of [25], we have considered it, expressed as

(24) $$J=2\pi \int dR\;\Sigma \left(R\right)R^{2}\sqrt{R\frac{\partial \Phi }{\partial R}}=\int dR\;Ry^{\prime }\sqrt{y}.$$

**Figure 4:**
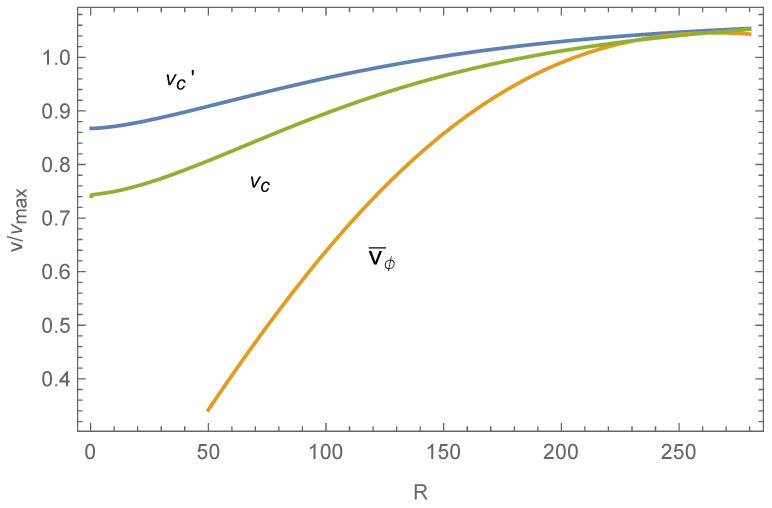
$v_{c}^{\prime }$ is calculated with Equation (24); $v_{c}$ and $\bar{v_{\phi }}$ are the results of Equation (28) with Equation (25).

**Figure 5:**
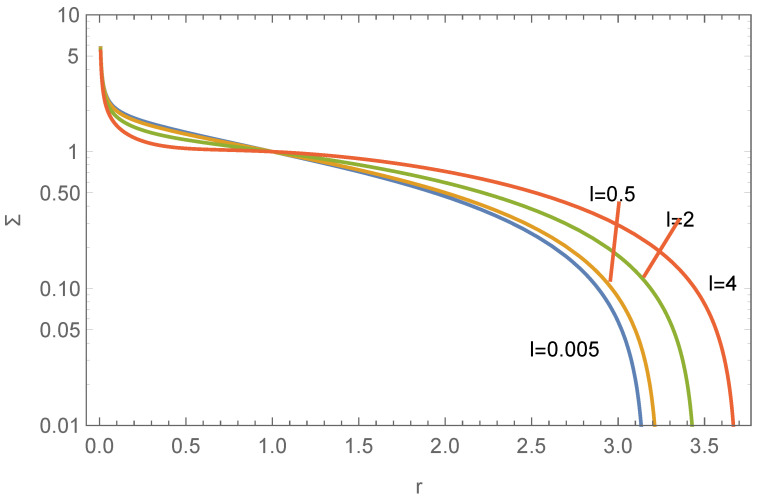
The influence of *l* on the surface density profile of the stellar disk, described by Equation (28). Parameters set as Σ(1)=p(1)=f(1)=γ=1, μ=0.05, b=0.0.

**Figure 6:**
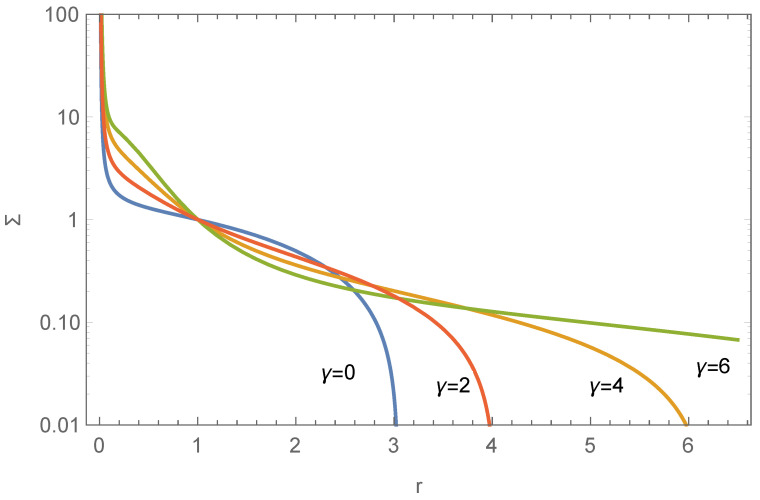
The influence of γ on the surface density profile of the stellar disk, governed by Equation (28). Parameters set as Σ(1)=p(1)=f(1)=l=1, μ=b=0.05. The spin parameter changes from 0.3188 to 0.1644 with increasing γ.

However, as evident from Figure 4, the circular velocity does not vary significantly across the entire disk, particularly not declining steeply in the central region, contradicting observations [31]. We adopt the form of $\bar{v_{\phi }}$, proposed by [32]:

(25) $$\bar{v_{\phi }}=\frac{\omega R}{1+{\left(R/R_{a}\right)}^{2}},$$

where $R_{a}$ in the original model is defined as $R_{*}$ here. Equation (25) indicates that the disk undergoes solid body rotation ($\bar{v_{\phi }}∼\omega R$) for $R\ll R_{a}$, and $\bar{v_{\phi }}$ decays as 1/R for $R\gg R_{a}$. With

(26) $$J=\int dR\;y^{\prime }R\bar{v_{\phi }}$$

and the variation

(27) $$\delta S_{t}=\delta S-\alpha \delta M-\beta \delta E-\Gamma \delta J=0,$$

the final dimensionless equation group describing the stellar disk is

(28) $$\begin{array}{ccc} {\left(\ln\left(p/\Sigma {\left(r\right)}^{2}\right)\right)}^{\prime } & = & \frac{b}{r^{2}}+\gamma \frac{2r}{{\left(1+r^{2}\right)}^{2}},\\ p^{\prime } & = & -\Sigma \left(r\right)\left(f\left(r\right)+\frac{\mu }{r^{2}}-\frac{lr}{{\left(1+r^{2}\right)}^{2}}\right),\\ f^{\prime }\left(r\right)+\frac{f(r)}{r} & = & \Sigma (r), \end{array}$$

where γ is determined by the total angular momentum, $l=\omega^{2}R_{*}^{2}\rho_{*}/P_{*}$, and other quantities are as defined in Equation (15). Irrespective of whether *r* is large or small, the term involving *l* tends to zero, thus having weak impact on the SBPs of the stellar disk, but from Figure 5 we can still find that the stellar disks with the same surface density but smaller spin have a slightly smaller radius, and l=0 is just the above case with no angular momentum. We set l=1 in this work. A dimensional analysis suggests that γ represents the ratio between the energy from particle’s random motion and orbital motion, implying an inverse correlation with the spin parameter, as confirmed by numerical calculations in Figure 6. This figure illustrates the effect of γ: with the same SAMD, a smaller γ (hence a larger spin parameter) corresponds to a shorter scale length of the exponential disk. This phenomenon can be understood by the following: let us consider a system of N stars with the total mass small compared to the central black hole, likely forming a disk with a large spin parameter; if we only increase the number or total mass of stars which perform the random motions with $\bar{v_{\phi }}=0$ so that the SAMD does not change, the system’s final radius will enlarge, its shape will become more elliptical, and the spin parameter of the stellar system will decrease. From the view of *J*’s definition (26), we can see that if the SAMD does not change, *J*’s (thus γ’s) increase will be caused by the increasing surface density (also can be seen in Figure 6), so the mass and energy of the disk will also significantly increase, and the spin parameter will not always increase as readers imagine. For instance, the dark matter halo and the disk are often assumed to share the same specific angular momentum (see page 505 of [33]), yet the radius of the larger spin disk is much shorter than that of the dark matter halo with a small spin.

Additionally, it is important to emphasize that the γ term cannot be neglected, especially for classical exponential or up-bending disks, to accurately fit the SBPs. At a sufficient distance, the terms involving *b*, γ, μ will vanish, and Equation (28) reverts to Equation (14) with μ=0, suggesting that the surface density profile will always exhibit a down-bending trend at large distances. Figure 3 shows that the central SBP of NGC3631 is better fitted by Equation (28). Figure 4 incidentally computes its circular velocity in Equation (28), indicating a decrease in the central region but not tending to zero.

## 4. Discussion and Conclusions

Building on our previous work on saddle-point entropy and following a similar approach to [26], we examined the impact of the central body alongside an improved specific angular momentum distribution. Our study, conducted in two dimensions, reveals that if the black hole’s potential also satisfies the 2D Poisson equation, only a limited number of disk SBPs can be described, suggesting a logarithmic profile. To more accurately represent the effects of the central body, we employed two methodologies: one is to persist with 2D calculations and consider $M_{•}$’s form of gravity similar to the Newtonian, i.e., Equation (15), and the other is to recalculate the whole variation in the 3D case, i.e., Equation (23). Both approaches indicate that the surface density behaves as $e^{r_{0}/r}$ and the energy constraint is satisfied. This implies that a perfectly 2D stellar disk is unlikely due to the necessity of constraining energy in reality. Additionally, for the perfect 2D system, the entropy can be globally maximized (see [34] or Equation (27) in [25]), but the entropy may be still a saddle point for the actual self-gravitating system in equilibrium. In the first approach, when $M_{•}$ is not excessively large, we derived analytical relationships between $M_{•}$, $M_{b}$, and the overall disk properties, pending further observational validation. Utilizing the model from [32], we further explored the influence of angular momentum on SBPs, finding that with the same surface density a smaller spin only slightly shortens the radius of the stellar disk; with the same SAMD, a larger spin more significantly restricts the disk’s extent. Our equation group also examines the effects of other parameters, aligning fundamentally with our physical understanding and offering improved explanations for SBPs, particularly in central regions. A distinctive aspect of our work is the prediction that the surface density profile will consistently exhibit downbending at a considerable distance from the center.

However, our study has limitations. We overlooked the black hole’s accretion and feedback, which might be crucial for the central region, although this feedback is not yet fully confirmed observationally; even recent studies suggest that the accretion onto a supermassive black hole is a multi-scale process [35,36], and we will conscientious study this effect in the future. We adopted a 2D gravitational law to align with flat rotation curves, but the central body’s gravity is proportional to $1/R^{2}$, indicative of a 3D model. While we can argue that the central body is modeled as a point, this remains a simplification. Furthermore, we propose that $R_{h}$ should represent the radius of the dark matter halo to maintain consistency with the 2D gravitational law, but the precise definition of $R_{h}$ remains an open question. For tri-exponential disks, our current approach does not systematically provide a better fit for their SBPs.

Despite these challenges, our work underscores the significant role of statistical mechanics in self-gravitating systems for understanding the interrelations between black holes, bulges, and disks. We aim to address these issues in future research. This work is intended to provide foundational insights, encouraging further contributions and ideas from the research community.

## Data Availability

The data of the SBP of NGC3631 are from [30], and in calculations we use the Mathematica 11 software.
